# Whole genome sequence of *Mycobacterium abscessus* NCVTCBAA1974 isolated from blue bull in India

**DOI:** 10.1128/mra.01167-25

**Published:** 2026-02-09

**Authors:** Indu Rani, Karuppusamy Shanmugasundaram, Harisankar Singha, Rajesh Kumar Vaid, Riyesh Thachamvalley, Bhupendra Nath Tripathi, Tarun Kumar Bhattacharya

**Affiliations:** 1 ICAR-National Research Centre on Equines 235426, Hisar, Haryana, India; 2 Sher-e-Kashmir University of Agricultural Sciences and Technology of Jammu, Jammu, Jammu and Kashmir, India; Rochester Institute of Technology, Rochester, New York, USA

**Keywords:** *Mycobacterium abscessus*

## Abstract

*Mycobacterium abscessus* is an opportunistic mycobacterium, frequently associated with non-tuberculous mycobacterial infections. Whole genome sequencing facilitates a comprehensive understanding of an organism’s genetic makeup and is crucial for therapeutics and disease control. Therefore, we present the whole genome sequence of *M. abscessus* with a genomic size of 4,781,464 bp and a GC content of 64.18%.

## ANNOUNCEMENT

Globally, the infection rates due to non-tuberculous mycobacteria (NTM) are increasing exponentially (1). NTM can cause infections in both humans and animals (2). Furthermore, NTM have intrinsic resistance to antimicrobials that are effective against *Mycobacterium tuberculosis* complex, making these infections notoriously difficult to treat. Among NTM, *Mycobacterium avium* complex and *Mycobacterium abscessus* are mainly responsible for most of the lung infections (3, 4). *M. abscessus* is an environmental opportunistic bacterium that causes a wide range of infections, such as lung, skin, and soft tissue infections in patients with underlying risk factors (5). *M. abscessus* infection is observed in both humans and animals, including dogs and cats (6). The disease transmission occurs through fomites and water systems (7). However, no strong evidence has been documented on animal-to-human transmission. Therefore, the precise transmission route and its zoonotic potential need further investigation. The genomic analysis of *M. abscessus* could provide valuable insights into its survival, antimicrobial resistance, and pathogenicity in the host.

Here we report the draft genome of an *M. abscessus* strain isolated from a fecal sample of a blue bull. For bacterial isolation, 5 g of fecal sample was homogenized with mortar and pestle and decontaminated with 10 mL of 0.75% hexadecylpyridinium chloride (HPC) (Sigma, USA). To remove the HPC residues, the pellet was washed thrice with phosphate-buffered saline (HiMedia, India) and suspended in 1 mL of sterile distilled water, and finally, 100 µL of the suspension was inoculated onto slants of Lowenstein-Jensen media and incubated at 37°C for 1–2 weeks. Subsequent to the incubation period, mature colonies were screened for positive mycobacterial growth by Ziehl-Neelsen staining. Genomic DNA was isolated from colonies using the Quick-DNA Fungal/Bacterial Miniprep Kit from Zymo Research, USA (D6005), according to the manufacturer’s protocol. The primer set *afb* for the 16S rDNA gene—F-5′−3′ GCGTGCTTAACACATGCAAGTC and R-5′−3′ TCCTCCTGATATCTGCGCATTC (8)—and *hsp65*—F 5′−3′ ACCAACGATGGTGTGTCCAT and R 5′−3′ CTTGTCGAACCGCATACCCT (heat shock proteins) (9)—was used to amplify a 650 and 439 bp segment, respectively, for PCR. The amplified products were PCR purified and sequenced commercially using the ABI 3730 Genetic Analyzer. Sequencing results showed ≥99% identity level when sequences were aligned with the *M. abscessus* reference strain using NCBI BLAST and finally confirmed the identity of the isolate as *M. abscessus*. For WGS, paired-end sequencing was performed using the Illumina NovaSeq 6000 platform, generating 43,767,208 read pairs (151 bp). The raw sequencing data were analyzed for quality assessment using FastQC (v0.12.1). Based on quality metrics, adapter trimming and filtering of low-quality reads were carried out using Trimmomatic (v0.39). High-quality reads were assembled *de novo* using SPAdes (v3.15.5). The resulting contigs were taxonomically classified using fIDBAC, and scaffolding was performed using Ragtag (v2.1.0). The genome was annotated by using the NCBI Prokaryotic Genome Annotation Pipeline (v6.10) (10).

The assembled genome of *M. abscessus* has a single contig with a total length of 4,781,464 bp, an average G + C content of 64.18%, and an assembly coverage of 1,989×. This genome consists of 4,597 protein-coding sequences, 46 transfer RNA genes, and 3 ribosomal RNA genes. No antimicrobial resistance testing was performed for this strain. However, to better characterize the strain, AMR testing needs to be performed in future studies.

## Data Availability

The *Mycobacterium abscessus* NCVTCBAA1974 strain genome sequence has been deposited in NCBI GenBank under accession number JBRFLY000000000. The BioProject can be found under the reference PRJNA1307395. The raw sequence data have been deposited in NCBI Sequence Read Archive under accession number SRR36699525.
